# Impact of diesel and biodiesel contamination on soil microbial community activity and structure

**DOI:** 10.1038/s41598-021-89637-y

**Published:** 2021-05-25

**Authors:** Eduardo K. Mitter, James J. Germida, J. Renato de Freitas

**Affiliations:** 1 Department of Food and Bioproduct Sciences, University of Saskatchewan, 51 Campus Drive, Saskatoon, SK S7N 5A8 Canada; 2 Department of Soil Science, University of Saskatchewan, 51 Campus Drive, Saskatoon, SK S7N 5A8 Canada

**Keywords:** Ecology, Microbiology, Molecular biology, Environmental sciences

## Abstract

Soil contamination as a result of oil spills is a serious issue due to the global demand for diesel fuel. As an alternative to diesel, biodiesel has been introduced based on its high degradability rates and potential for reducing of greenhouse gases emissions. This study assessed the impacts diesel and biodiesel contamination on soil microbial community activity and structure. Our results suggest higher microbial activity in biodiesel contaminated soils and analysis of PLFA profiles confirmed shifts in microbial community structure in response to contamination. High-throughput 16S rRNA amplicon sequencing also revealed a lower bacterial richness and diversity in contaminated soils when compared to control samples, supporting evidence of the detrimental effects of hydrocarbons on soil microbiota. Control samples comprised mostly of Actinobacteria, whereas Proteobacteria were predominantly observed in diesel and biodiesel contaminated soils. At genus level, diesel and biodiesel amendments highly selected for *Rhodococcus* and *Pseudomonas* spp., respectively. Moreover, predicted functional profiles based on hydrocarbon-degrading enzymes revealed significant differences between contaminated soils mostly due to the chemical composition of diesel and biodiesel fuel. Here, we also identified that Burkholderiaceae*, Novosphingobium, Anaeromyxobacter, Pseudomonas* and *Rhodococcus* were the main bacterial taxa contributing to these enzymes. Together, this study supports the evidence of diesel/biodiesel adverse effects in soil microbial community structure and highlights microbial taxa that could be further investigated for their biodegradation potential.

## Introduction

Petroleum derived diesel fuel is a major source of energy throughout the world and one of the most widespread soil contaminants^1,2^. Diesel contamination causes significant impacts on soil properties that can lead to water and oxygen deficits as well as shortage of available forms of nutrients such as nitrogen and phosphorus^3^. Thus, due to the high demand for diesel and the decline of fossil fuel reserves, less-polluting and renewable fuel sources such as biodiesel are currently being investigated^4^.

Biodiesel derived from vegetable oils are widely encouraged in several countries as an alternative to non-renewable petroleum based products^5,6^. Biodiesel fuel is produced by trans-esterification of fatty acids with an alcohol (usually methanol) in the presence of a catalyst, and it can ultimately replace diesel partially or completely^7^. The environmental benefits of biodiesel includes lower emissions of particulate matter and greenhouse-effect gases, and no release of sulfur and volatile aromatic compounds into the atmosphere^5^. Also, recent studies demonstrate that biodiesel is more readily degraded by microorganisms than diesel, since it consists of alcohol esters of short chain fatty acids, which are compounds that exist naturally in the environment^8^. However, diesel or biodiesel oil spills may cause shifts in soil microbial community structure which can lead to greater impacts on soil physical–chemical proprieties and ecosystem functioning.

Microorganisms are key determinants of soil physical, biological and chemical characteristics, biogeochemical cycling and other terrestrial ecosystem functions^9^. Hence, the sensitivity of soil microbial community structure to ecosystem disturbance may be an indicator of soil pollution and soil health^10^. However, despite the importance of microbial community composition to soil ecosystem functioning, recent studies have mostly focused only on diesel bioremediation strategies by bioaugmentation^11^ or biostimulation^1,12^. Studies by Woźniak-Karczewska et al.^13^ assessed shifts in soil microbial community structure due to contamination diesel/biodiesel blends, but only after bioaugmentation with a microbial consortia. Therefore, to the best of our knowledge, this is the first study to compare the effects of long-term biodiesel and diesel natural attenuation on soil microbial community structure using two culture independent techniques (phospholipid fatty acid analysis and high-throughput 16S rRNA amplicon sequencing).

The main objective of this study was to evaluate the impacts of diesel and a canola-derived biodiesel fuel on soil microbial community activity and composition. We monitored microbial activity by CO_2_ production within the first 5 weeks of upon contamination and assessed shifts in microbial community structure after a 1-year incubation. Phospholipid fatty acid (PLFA) analysis was used to detect more immediate changes in microbial community structure in dominant bacterial taxa. We also used high throughput DNA sequencing for an in-depth taxonomic assessment in these soils and metagenomic functional modelling to predict its biodegradation potential. We hypothesized that both fuels would significantly impact soil microbial communities by altering its diversity, community structure/activity while selecting for different taxa capable of degrading these contaminants.

## Results

### Soil chemical analysis and microbial activity

**Figure 1:**
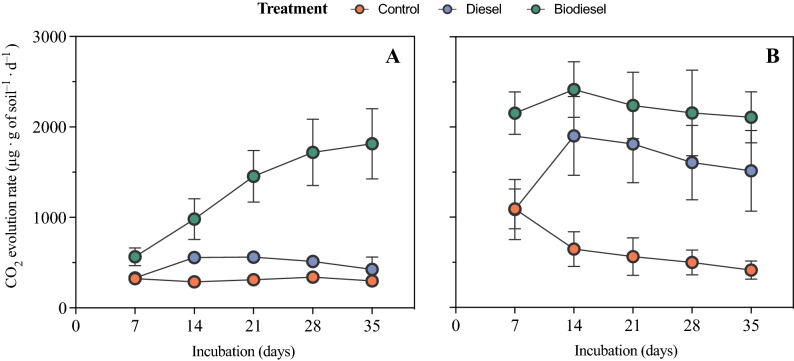
Soil microbial activity (CO_2_ evolution) measurements in an upper (**A**) and lower (**B**) slope soil under three different treatments (control biodiesel and diesel) after 35 days. Error bars represent standard deviations (n = 5).

Soil chemical analyses exhibited differences among the two soils collected (Table S1). The upper slope soil had a higher pH, whereas the soil collected at the lower slope indicated higher organic matter, available N, S, P and K. Analysis of microbial CO_2_ evolution also detected differences between the two soils, yet a similar tendency was observed among treatments (Fig. 1). For example, biodiesel amended soils exhibited the highest CO_2_ production followed by diesel and control samples. After a 1-year incubation, results for total nitrogen (TN) revealed no significant differences based on treatment (Table S2). However, total organic carbon (TOC) and total carbon (TC) were significantly higher in both soils amended with biodiesel. In addition, diesel contaminated soils had the highest rates of inorganic carbon (IC) content in upper slope soils.

### PLFA analysis

Analysis of PLFA biomarkers revealed that microbial community structure was primarily affected by treatment (i.e., diesel or biodiesel amendment) followed by soil type (i.e., upper or lower slope) (Table S3). With the exception of fungal PLFAs, significant differences were detected between treatments for all biomarkers (*p* < 0.05). For example, Gram-positive (G+) bacteria biomass was highest on diesel treatments in lower slope soils in both absolute and relative abundance (mol%). Compared to control treatments, biodiesel addition stimulated Gram-negative (G−) bacteria, but inhibited G+ bacteria in both soils (Table S3). Similarly, biodiesel treatments exhibited the highest values of total PLFAs (*p* < 0.05), which varied from 49.6 to 44.2 nmol·g^−1^ on soils in the upper and lower slope, respectively (Fig. S1).

**Figure 2:**
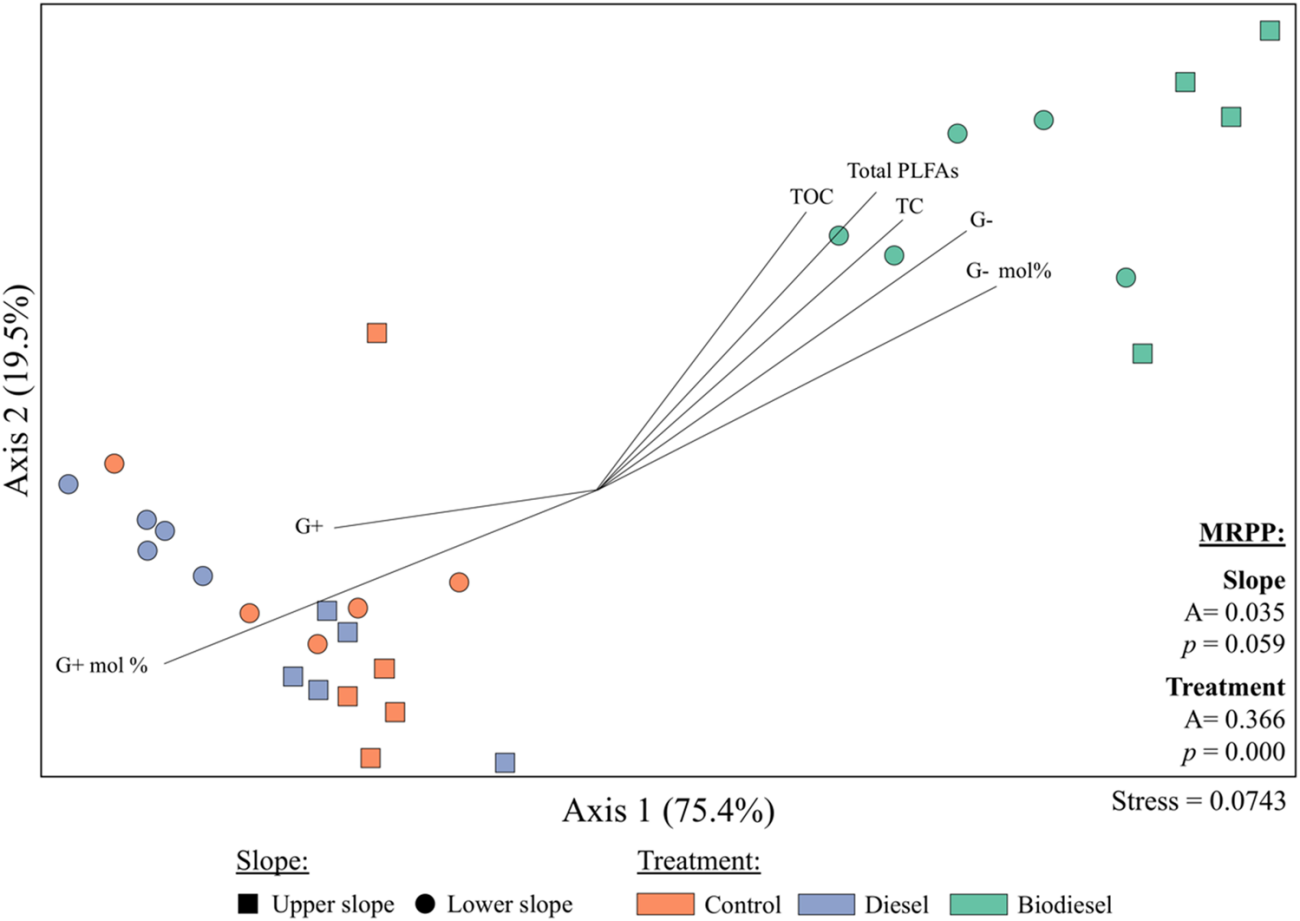
Two-dimensional solution of non-metric multi-dimensional scaling (MDS) ordination analysis and multiple response permutation procedure (MRPP) of PLFA profiles from an upper slope and lower slope soils under three different treatments (control, diesel and biodiesel). Percentage values in axes represent the percentage of variance explained by each axis. Vectors indicate direction and strength of relationships of specific PLFA groups and environmental variables (not included in matrix distance calculations). G+ and G− represent absolute Gram positive and and Gram negative biomarker abundance, respectively. Relative abundance G+ and G− biomarkers are shown as mol%. *TOC* = total organic carbon and *TC* = total carbon. The full list of soil parameters analyzed and specific PLFA biomarkers by treatment and slope are provided in Tables S2 and S3, respectively.

Non-metric multi-dimensional scaling (MDS) ordination from PLFA profiles indicated clusters by treatment within microbial community profiles that were confirmed by multi-response permutation procedure (MRPP) analyses (*p* < 0.05) (Fig. 2). Here, two clustering groups were identified including: (i) biodiesel amended soils that positively correlated with soil carbon (TC and TOC), total PLFAs and G− bacteria; (ii) diesel and control treatment groups that exhibited positive correlations with G+ bacteria (i.e., absolute and relative abundance).

### High-throughput 16S rRNA amplicon sequencing

**Figure 3:**
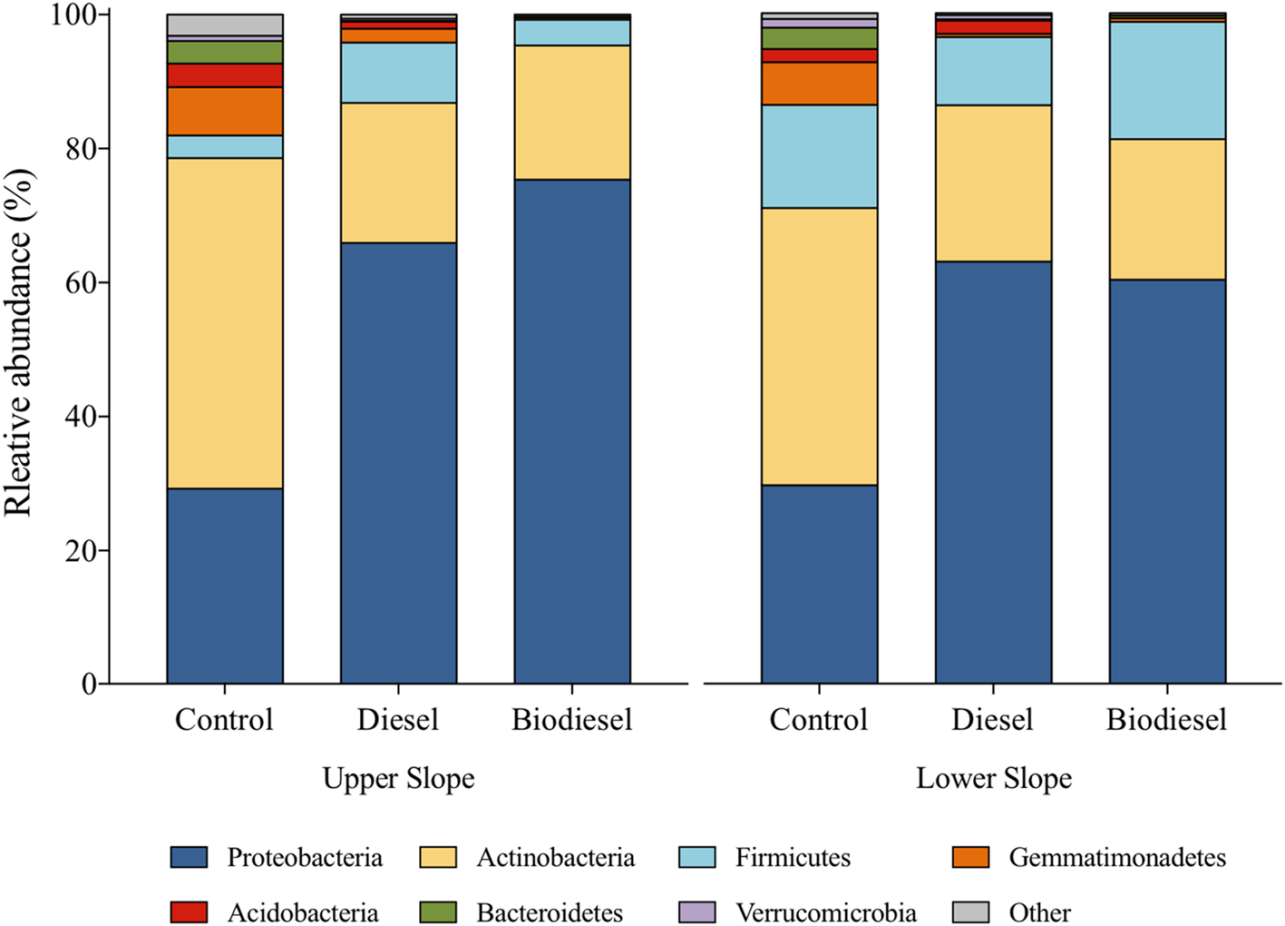
Analysis of bacterial phyla of bacterial communities in an upper slope and lower slope agricultural soil under three different treatments (control, diesel and biodiesel) (n = 5) after 1 year of incubation.

High-throughput sequencing analysis of the V4 region of the 16S-rRNA gene indicated a recovery of 458,158 high quality sequences and 1716 unique sequences in 30 soil community samples. A total of 20 phyla was detected in the dataset, in which only five distinct phyla comprised approximately 90% of the profile. Proteobacteria and Actinobacteria were the most abundant phyla considering all samples analyzed (Fig. 3). Control soils exhibited a dominance of Actinobacteria (> 40%) while diesel and biodiesel contaminated soil had a high abundance of Proteobacteria (> 60%). Other phyla such as Gemmatimonadetes and Firmicutes corresponded less than 20% and 10% of all profiles, respectively. Correlation analysis between soil chemical parameters and relative abundance of bacterial profiles revealed significant associations at phylum, class and family levels (Table S4). Most significant correlations were observed with TOC and TC. For example, our analyses indicate positive correlations between TC and Proteobacteria (*r*_*s*_ = 0.69, *p* < 0.01)*;* yet, negative correlations with Actinobacteria (*r*_*s*_ =  − 0.69, *p* < 0.01), Bacteroidetes (*r*_*s*_ =  − 0.59, *p* < 0.01) and Gemmatimonadetes (*r*_*s*_ =  − 0.79, *p* < 0.01). Similarly, most significant correlations at lower taxonomic levels (i.e., class and order) were detected with soil carbon.

Venn diagram of amplicon sequence variants (ASV) in each treatment revealed a total of 758 (44.2%), 384 (22.4%) and 182 (10.6%) that were unique to control, diesel and biodiesel, respectively (Fig. S2). In addition, only 130 ASVs were common to all profiles, thus representing 7.6% of the total number of ASVs. Alpha diversity indexes (i.e., chao1 richness, Shannon and Simpson diversity) showed significant differences between treatments and slope (Table 1). Overall, higher richness and diversity were observed in control samples for both soil slopes analyzed. In biodiesel contaminated soils, the lowest alpha diversity indexes were detected in upper slope soils; whereas in diesel treatments, the lowest alpha diversity indexes were observed in lower slope soils. Spearman’s rank correlations with soil chemical parameters also revealed overall negative correlations between alpha diversity indexes and soil carbon (TOC and TC). No significant correlations were observed between diversity indexes and soil inorganic carbon or soil nitrogen (Table S5).

**Table 1:** Alpha diversity indexes of bacterial communities in an upper slope and a lower slope soil under three different treatments (control, biodiesel and diesel).

	chao1	Shannon	Simpson
**Upper slope**			
Control	261.4^ab^	7.33^a^	0.99^a^
Diesel	201.3^abc^	5.60^bc^	0.92^abc^
Biodiesel	122.0^c^	4.14^c^	0.73^c^
**Lower slope**			
Control	265.0^a^	7.15^ab^	0.98^ab^
Diesel	126.4^c^	4.10^c^	0.82^bc^
Biodiesel	166.8^bc^	5.36^c^	0.92^abc^
***p* value**			
Slope (S)	0.333	0.295	0.920
Treatment (T)	< 0.01	< 0.01	0.014
S* T	< 0.01	< 0.01	< 0.01

Different letters indicate significant differences (Tukey HSD *p* < 0.05) (n = 5) after 1 year of incubation.

**Figure 4:**
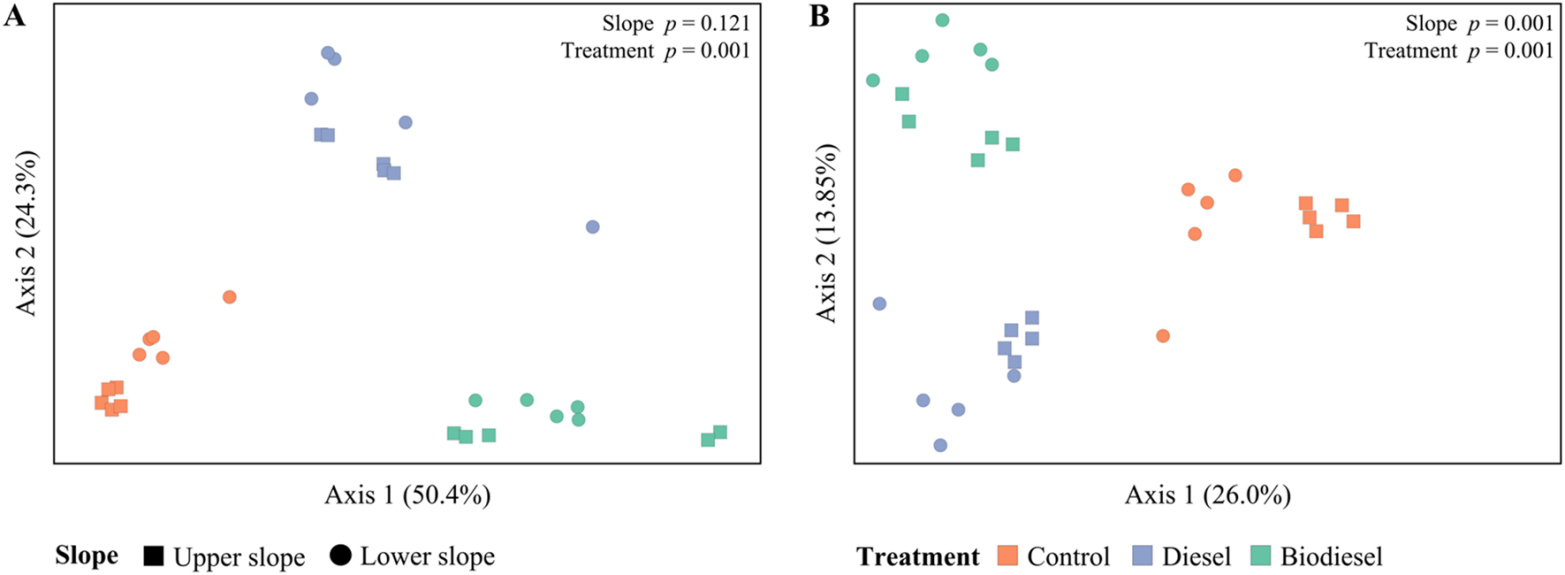
Principal coordinate analysis (PCoA) based on (**A**) weighted and (**B**) unweighted unifrac distances between soil samples for treatment and slope (n = 5). Adonis test (999 permutations) was used to determine differences in community composition between groups (*p* values shown).

**Figure 5:**
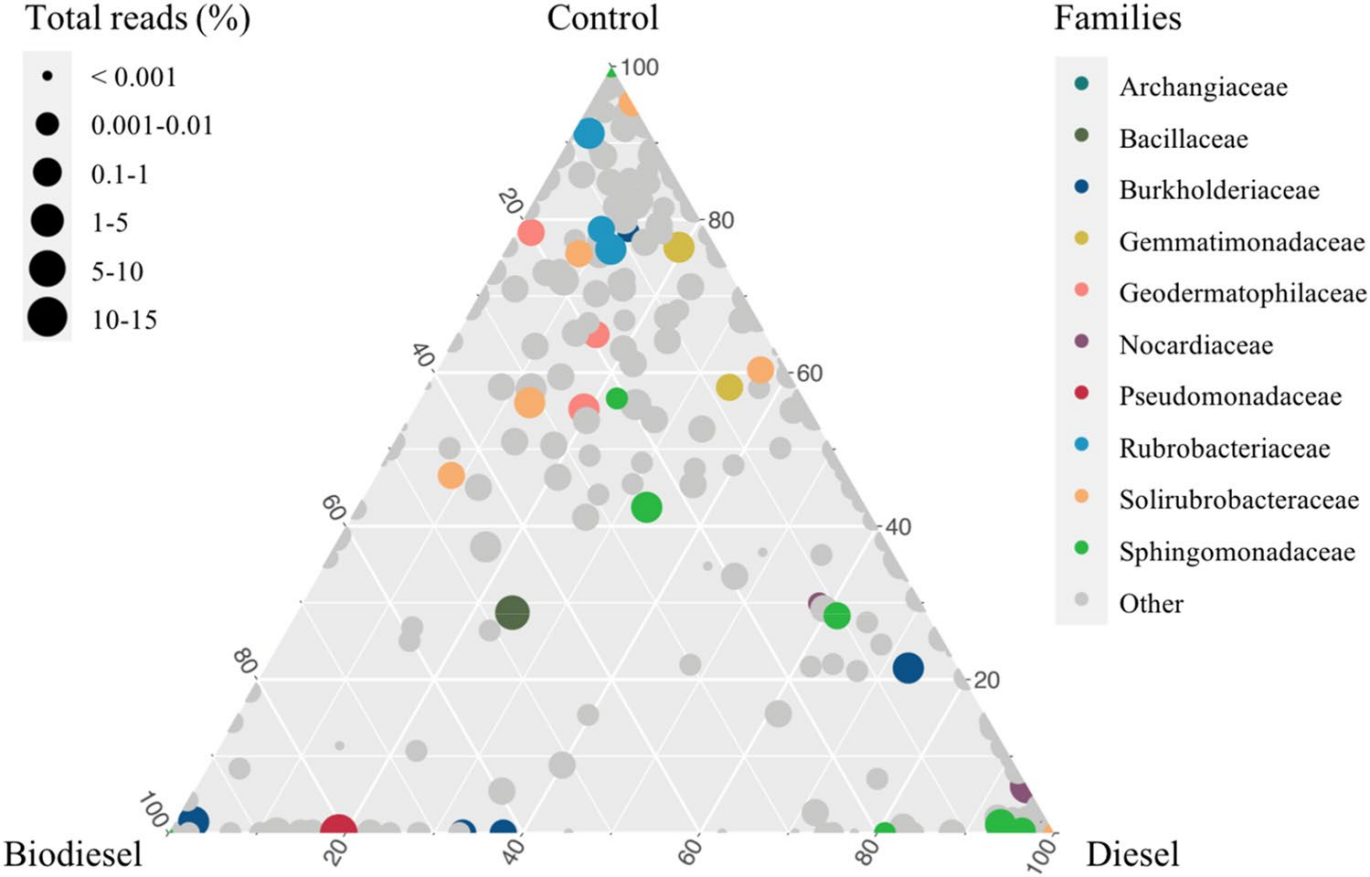
Ternary plot representing the relative occurrence of bacterial genera (circles) in soils under three different treatments (control, diesel and biodiesel). Genera enriched in different treatments are colored at family level and circle size is proportional to their abundance in the community. This figure was generated using the ‘ggtern’ package in R.

Analysis of ß-diversity using principal coordinate analysis (PCoA) revealed a clear separation in16S rRNA profiles by treatment (*p* = 0.001) (Fig. 4), and significant differences between slope positions (*p* = 0.001) when considering unweighted unifrac distances (Fig. 4B). This evidence was further analyzed using a ternary plot at genus level, color coded by the most abundant families in the dataset (Fig. 5). Here, genera from the family Gemmatimonadaceae and Rubrobacteriaceae were more closely associated with control samples, whereas members of the family Burkholderiaceae were mostly detected in both diesel and biodiesel contaminated soils.

**Figure 6:**
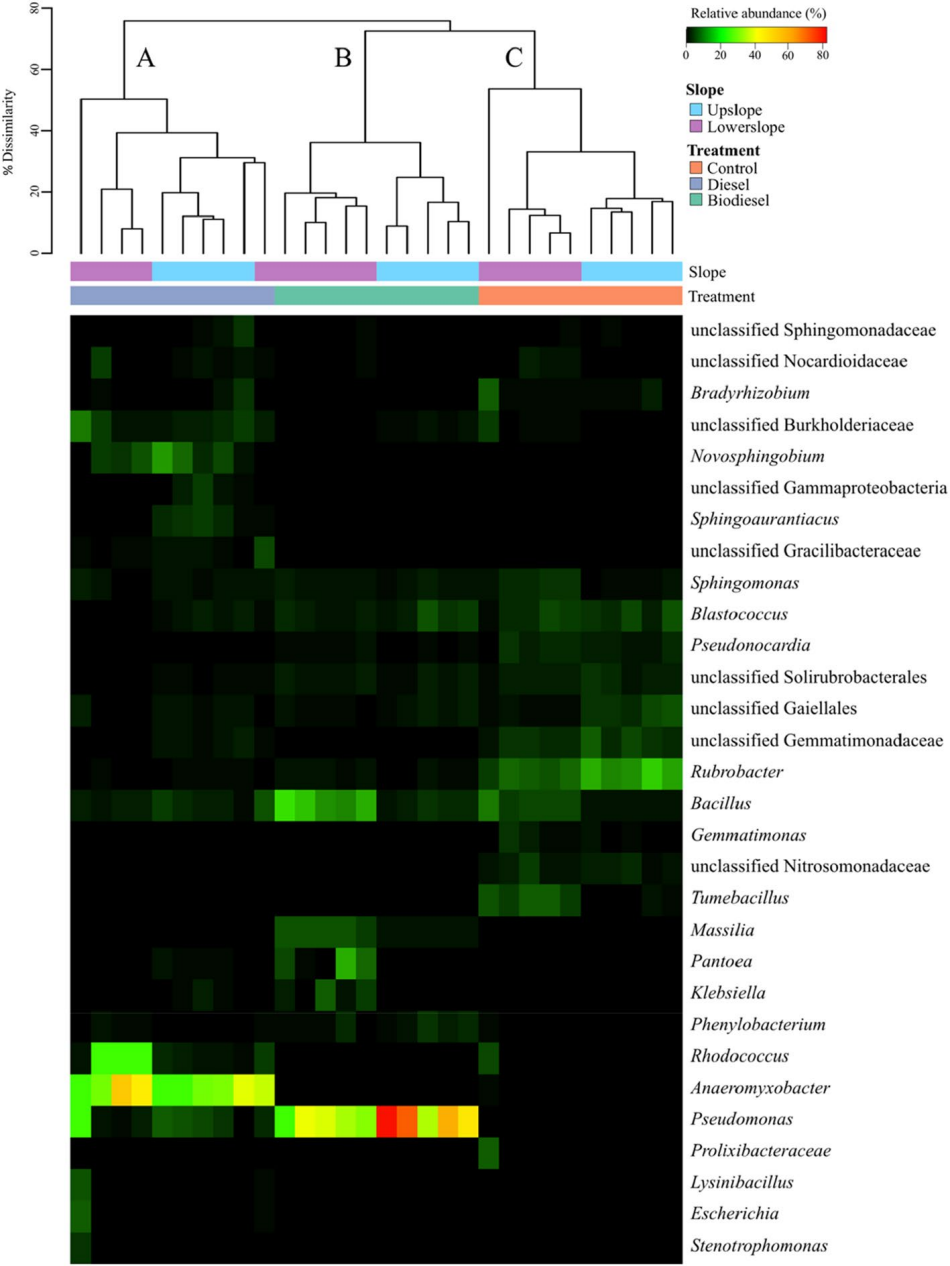
Heatmap based on relative abundance of soil bacterial communities. Columns represent samples, rows represent genera that are 4% most abundant in at least one sample. Clustering of samples (top) are based on genera co-occurrence by Bray–Curtis dissimilarity (n = 30). This figure was generated using the vegan package ‘vegdist’ function in R.

To assess the main genera driving differences in microbial community structure after diesel and biodiesel amendment, a heatmap based on Bray–Curtis dissimilarity was generated in order to compare bacterial profiles (Fig. 6). Our analysis confirmed that these profiles clustered mainly by treatment where three main clusters (A–C) were observed after a 65% dissimilarity cut off. Cluster A (left to right) corresponded to diesel amended soils, which consisted mainly of *Anaeromyxobacter* (31.5%), *Rhodococcus* (8.67%), *Pseudomonas* (5.2%), *Novosphingobium* (4.8%) and unclassified genus from the family Burkholderiaceae (3.7%). *Anaeromyxobacter* was the indicator genus driving these differences in which it could comprise up to 50% of profiles. Cluster B consisted exclusively of biodiesel samples, which were driven by a high abundance of *Pseudomonas* (comprising up to 76% of in some profiles and on average 43%). Additional genera such as *Bacillus* (8.2%), *Massilia* (4.0%), *Blastococcus* (3.1%) and *Pantoea* (3.1%) were also included in cluster B (Fig. 6). Moreover, we also identified a third cluster (Cluster C) consisting only by control samples, in which no particular genera corresponded to more than 15% of the profile. In this cluster, the most abundant genera detected were *Rubrobacter* (9.9%), an unclassified genus from the family Gemmatimonadaceae (4.2%), *Bacillus* (4.2) *Blastococcus* (4.2%) and *Tumebacillus* (3.4%).

Relative abundance of the most abundant taxa between diesel and biodiesel treated soils was also compared using Welch’s t-test (*p* < 0.05) (Fig. S3). A total of 27 bacterial genera was significantly different between these soils. Whereas diesel treatments had a higher abundance of *Anaeromyxobacter* and *Rhodococcus*, soil amendment of biodiesel fuel favoured *Pseudomonas* ssp.

**Figure 7:**
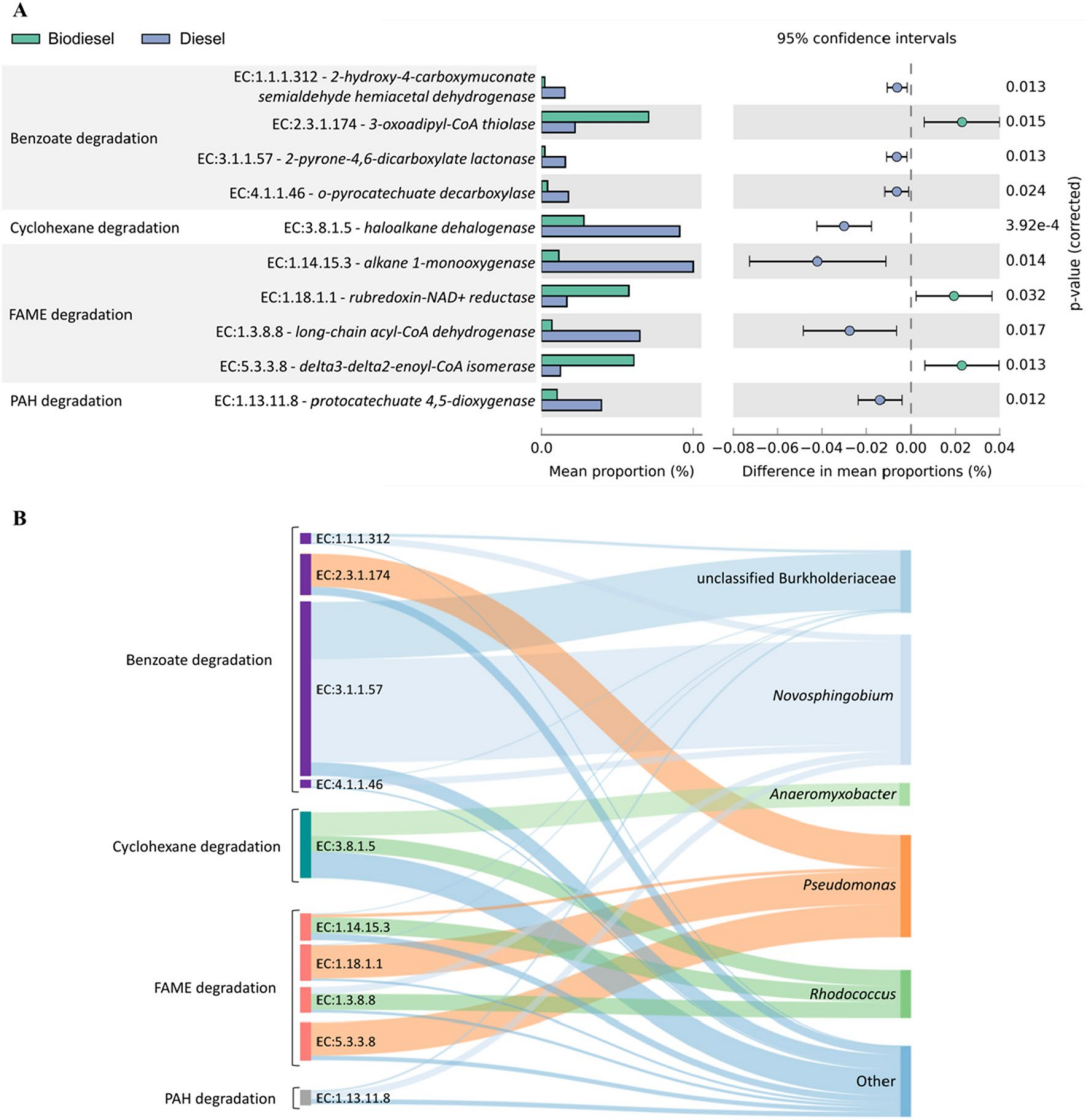
(**A**) PiCRUSt2 predicted hydrocarbon degrading enzymes in biodiesel and diesel amended soils. Extended bar plots represent only statistically significant microbial enzymes between treatments based on Welch’s t-test (*p* < 0.05) with a minimum of 10 reads per sample. (**B**) Sankey diagram indicating the relative contribution of predicted hydrocarbon degrading enzymes (ECs) by bacterial taxa. Vertical nodes are proportional to size.

Functional modelling using PICRUSt2 revealed 411 MetaCyc microbiome metabolic pathways^14^ in 1716 ASVs. Here, we initially compared the functional profiles between contaminated (diesel and biodiesel) and control soils (Fig. S4). Our results revealed that whereas both groups had a high abundance of biosynthesis pathways, degradation pathways abundance was significantly higher in contaminated soils (*p* < 0.05). For example, contaminated soils had higher abundance of metabolic pathways in propanoate degradation, octane oxidation and sugar degradation. Therefore, we analyzed the predicted metabolic profiles of contaminated soils targeting for enzymes associated with these pathways (Fig. 7A). More specifically, we targeted enzymes in the KEGG ortholog reference pathways for fatty acid (FAME) degradation (ko00071), metabolism of xenobiotics by cytochrome P450 (ko00980), polycyclic aromatic hydrocarbon degradation (ko00624), chlorocyclohexane and chlorobenzene degradation (ko00361), toluene degradation (ko00623) and benzoate degradation (ko00362). With the exception of 3-oxoadipyl-CoA thiolase (EC:2.3.1.174), our results suggest an overall higher proportion for enzymes involved in the benzoate, cyclohexane and polycyclic aromatic hydrocarbon (PAH) degradation pathways in diesel treatments (*p* < 0.05). Moreover, predicted fatty acid degradation enzymes such as alkane 1 monooxygenase (EC:1.14.15.3) and long-chain acyl-CoA dehydrogenase (EC:1.3.8.8) were also higher in diesel amended soils when compared to biodiesel. Yet, soils amended with biodiesel indicated higher abundance of rubredoxin-NAD + reductase (EC:1.18.1.1) and delta3-delta2-enoyl-CoA isomerase (EC:5.3.3.8) associated with the fatty acid degradation.

Based on predicted functional profiles, we also evaluated the taxa contribution of hydrocarbon degrading enzymes (Fig. 7B). Our results suggest a high contribution of *Novosphingobium* and an unclassified genera of the family Burkholderiaceae in benzoate degradation (i.e., EC:1.1.1.312, EC:3.1.1.57 and EC:4.1.1.46). Moreover, *Pseudomonas* spp. also contributed to benzoate degradation with more than 50% of the abundance of 3-oxoadipyl-CoA thiolase (EC:2.3.1.174). In cyclohexane degradation, more than 50% of the predicted haloalkane dehalogenase (EC:3.8.1.5) was attributed to the presence of *Anaeromyxobacter* and *Rhodococcus* spp. Fatty acid degradation enzymes were most exclusively represented by *Pseudomonas* and *Rhodococcus* spp.*,* whereas bacterial taxa such as Burkholderiaceae and *Novosphingobium* highly contributed to the enzyme protocatechuate 4,5-dioxygenase (EC:1.13.11.8) in PAH degradation. Therefore, after 1-year incubation, our results suggest that soil contamination with diesel and biodiesel led to different impacts not only in microbial community structure, but also in potential functional profiles associated with hydrocarbon degradation.

## Discussion

In this study, we provide a detailed assessment on the effects of diesel and biodiesel amendments in soil. We first monitored microbial activity upon the first 5 weeks of contamination followed by a characterization of microbial community structure and microbiome functional prediction after a 1-year incubation. Microbial activity, monitored by CO_2_ production, in biodiesel- and diesel-contaminated soils confirms the ability of microorganisms to degrade and use these compounds as carbon sources^8^. In turn, microbial respiration of control samples is related to the response of the microbial activity to basic soil nutrients in the absence of organic amendments. In our study, contaminated soils had an overall increase in microbial activity after 14 to 21 days followed by a decrease after 21 days, which may indicate a depletion of the substrate. Studies conducted by Silva et al.^8^ suggest that biodiesel amendment in soils resulted in the highest respiration rates, which confirms that biodiesel is more easily biodegradable than diesel. Similar results were also observed in our study as diesel-contaminated soils indicated the lowest microbial activity amongst amended soils. Lapinskiene et al.^15^ also observed similar results suggesting that diesel is more resistant to microbial decomposition than biodiesel. According to Schiewer et al.^16^, biodiesel degradation is typically faster than diesel, and biodiesel addition has even been used to stimulate hydrocarbon degradation in contaminated sands. After a 1-year incubation, TOC and TC content in our study was higher in treated soils since hydrocarbon contamination is known to increase total carbon content in soil^17^. Unexpectedly, we also detected a change of soil inorganic carbon in these soils, especially in soils amended with diesel fuel. Soil inorganic carbon, carbonates (HCO_3_^−^ and CO_3_^2−^) primarily associated with calcium and magnesium, are mostly affected by soil carbon dioxide, pH, Ca^2+^ content and water^18^. Previous studies on diesel contaminated soils found higher degradation rates in carbonate-rich soils^19^ and suggested that the CO_2_ produced by diesel mineralization could result in the formation of soil carbonates^20^.

Microbial community structure analyses were conducted using culture independent phospholipid fatty acid analysis (PLFA) and high-throughput 16S rRNA amplicon sequencing. We used PLFAs in addition to a nucleic acid based method (i.e., 16S rRNA amplicon sequencing), as phospholipids found in cell membranes of all living organisms and are rapidly degraded upon their death^21^. Hence, this analysis provides a measure of viable community biomass and structure. Significant differences of PLFA profiles where observed in both soil slopes and treatments; however, most differences were observed among treatments. In our study, microbial community profiles mainly clustered by treatments in which two distinct groups were identified: (i) soils amended with biodiesel, (ii) diesel and control treatment groups. Margesin et al.^22^ analyzed soil PLFA profiles based on total petroleum hydrocarbons (TPH) and observed a significant increase in the Gram-negative populations in high TPH amended-soils. Our results also indicated that soils amended with biodiesel stimulated the abundance of Gram-negative bacteria. However, diesel treatments and control samples exhibited the lowest amounts of total PLFAs, which may suggest that unlike biodiesel, diesel is not being metabolized at the same rate by bacterial communities, and therefore no increase in microbial abundance was observed. According to Margesin et al.^22^, G− bacteria are r-strategists and can rapidly grow under substrate-rich conditions. In our study, MDS analysis also revealed that an increase in TC and TOC content were highly associated with biodiesel treatments (Fig. 2). Similar results were observed by Owsianiak et al.^23^ using diesel/biodiesel blends as a carbon source for bacterial consortia. This study reported that higher biodiesel content in fuel blends led to greater microbial biomass, thus supporting evidence that biodiesel is a favored carbon source over diesel. However, degradation of PLFAs upon cell death is significantly faster than other cell components such as DNA, RNA, and proteins^24^. For this reason, PLFA analysis has long been used as a sensitive tool to detect community shifts in response to changing environmental conditions^25^. Yet, many fatty acids are common to different microorganisms^26^ and therefore we used high-throughput 16S rRNA amplicon sequencing to overcome these limitations.

High-throughput sequencing revealed that soil contamination with diesel and biodiesel affected bacterial profiles considerably. Actinobacteria, which were the most abundant phylum in control samples, play an important role in nutrient cycling due to their ability to metabolize complex organic matter^27^. In contrast, a high abundance of Proteobacteria was observed in diesel and biodiesel contaminated soils. Proteobacteria are known for their ability to utilize aliphatic and aromatic compounds, hence an increase in their abundance is often noted in hydrocarbon-amended soils^7,28,29^. Additionally, positive correlations between Proteobacteria and soil total carbon was observed in our study, as Proteobacteria are thought to respond positively to carbon and nutrient inputs in soil^30^. Possibly, the increased soil carbon levels due to biodiesel addition may have selected for bacteria that are able to utilize this amendment as a carbon source. Although Actinobacteria and Proteobacteria comprised most of the bacterial profiles in our dataset, we also observed an increased abundance of Firmicutes in contaminated soils. Firmicutes play a major functional role in the decomposition of plant polymers, yet a broad metabolic activity in aromatic and/or aliphatic hydrocarbons is rare among this phylum^31^. Moreover, some thermophiles such as environmental spore-forming *Geobacillus* and *Bacillus* strains, both members of the phylum Firmicutes, are known to inhabit hydrocarbon-impacted environments^32,33^.

In addition to bacterial community structure at phylum level, 44% of ASVs in our dataset were unique to control samples (Fig. S2). In fact, we also detected a significant reduction in bacterial richness and diversity in contaminated soils, thus suggesting the selection for specific bacterial consortia. Similar results were reported by Sutton et al.^28^, in which the presence of diesel contributed significantly to explaining shifts in soil microbial community structure. According to Bundy et al.^34^, hydrocarbon contamination often selects for reduced numbers of generalists and catabolically-versatile bacterial species. Similarly, PCoA analysis of bacterial profiles in our study indicated significant differences between treatments. Here, we observed clustering regions with a low variability between samples such as in biodiesel amended soils, and a high variability in control and diesel treatments.

Supporting the evidence of the selection of a few bacterial taxa in diesel and biodiesel contaminated soils, control soils mostly consisted of members from the family Gemmatimonadaceae and Rubrobacteriaceae, whereas Burkholderiaceae were more associated with contaminated soils. Members of the family Burkholderiaceae have been detected in the crude oil samples^35^, and many species of *Burkholderia*, such as *B. cepacia* are known to biodegrade hydrocarbons^36,37^.

Analysis of bacterial profiles at genus level revealed that *Anaeromyxobacter, Rhodococcus, Pseudomonas* and *Bacillus* are the main genera driving differences in microbial community structure in contaminated soils. In particular, our data suggests a high abundance of *Anaeromyxobacter* in diesel amended soils. The genus *Anaeromyxobacter* comprises of facultative anaerobic myxobacterium and have been found in hydrocarbon contaminated soils^38^. In addition, diesel-contaminated soils also indicated the presence of *Rhodococcus* spp., with an average relative abundance of 10%. Due to their hydrophobic cell surfaces, and their inherent ability to degrade a broad range of organic compounds and to produce biosurfactants, *Rhodococcus* are potential candidates for hydrocarbon biodegradation in soils^39^. In fact, Lee et al.^40^ reported that the inoculation of *Rhodococcus* sp., combined with mycolic acid as synthetic surfactant, significantly enhanced soil diesel biodegradation. While *Anaeromyxobacter* and *Rhodococcus* were the most abundant organisms in diesel contaminated soils, both biodiesel- and diesel-amendments favored the presence of *Pseudomonas* spp. Numerous studies reported that *Pseudomonas* are able to degrade naphthalene^41^, phenanthrene^42^, diesel^43^ and biodiesel^44^. According to Taccari et al.^45^, *Pseudomonas* spp. produce biosurfactants that may contribute to the desorption and degradation efficiency of petroleum derived hydrocarbons. In addition to *Pseudomonas* spp., biodiesel amended soils also exhibited a dominance of *Bacillus* spp. As Gram-positive, endospore-forming bacteria, *Bacillus* spp. exhibit a wide range of physiological abilities which includes adaptation to biodiesel-diesel contamination^46^ and active biodiesel degradation^47^. Differently from biodiesel- and diesel-contaminated soils, *Rubrobacter*, a known *Actinobacteria* well adapted for semi-arid soils^48^, was highest in control samples. In studies assessing soil contamination by hydrocarbons, a high abundance of genera from the phylum *Actinobacteria* have been previously reported in uncontaminated soil samples by Wollherr et al.^49^. Bell et al.^30^ also found negative correlations between *Actinobacteria* and soil hydrocarbon concentrations after diesel contamination.

Microbial profiling based on 16S rRNA is a key tool to analyzed changes in microbial community structure, but it lacks to provide direct evidence of their functional capabilities. Therefore, PICRUSt2 provides an opportunity to predict functional profiles based on 16S rRNA and it has been previously used to assess hydrocarbon-degrading potential^50,51^. Using the PICRUSt2 pipeline, we detected a higher abundance of metabolic pathways in propanoate degradation, octane oxidation and sugar degradation in contaminated soils. In particular, mean proportions of the octane oxidation pathway was much higher in these treatments when compared to control soils. This pathway describes organisms capable of using intermediate chain length n-alkanes (C6 to C12) as an energy source^14^. The alkane hydroxylase system is a key component of this pathway that in introduces molecular oxygen in the terminal carbon atom of hydrocarbon compounds to form primary alcohols^52^. Hence, PICRUSt2 analysis suggest that bacterial communities in soils contaminated with diesel and biodiesel developed specific mechanisms to adapt their metabolic pathways to hydrocarbon degradation. Moreover, profiles in contaminated soils also indicated a higher abundance of proteinogenic amino acid and vitamin biosynthesis. Similar results were observed by Mukherjee et al.^53^ in petroleum hydrocarbon contaminated sites, in which these authors attributed to a wide range of functions such as stress tolerance and redox responses. Therefore, based on the evidence of high proportions of predicted propanoate degradation, octane oxidation and sugar degradation pathways in contaminated soils, we focused our next analysis on specific groups of hydrocarbon degrading enzymes within these samples.

PICRUSt2 analysis revealed, with the exception of 3-oxoadipyl-CoA thiolase (EC:2.3.1.174), a higher abundance of enzymes associated with aromatic compound degradation (i.e., benzoate, cyclohexane and PAH degradation) predicted in diesel contaminated soils. For example, enzymes such as protocatechuate 4,5-dioxygenase (EC:1.13.11.8) and haloalkane dehalogenase (EC:3.8.1.5) both act on aromatic compounds. Protocatechuate 4,5-dioxygenase is a well-known oxidoreductase that catalyze the cleavage of the aromatic ring on aromatic compounds with the insertion of two oxygen atoms^54^. Haloalkane dehalogenases; however, catalyse the hydrolysis of halogenated alkanes where the halogen functional group is replaced with a hydroxyl group^55^. Most likely, a higher abundance of aromatic compound degradation enzymes in these soils are due to the chemical composition of diesel fuel. Diesel is a complex mixture of hydrocarbons (8–26 carbon atoms) which includes aromatic hydrocarbons (23.9%) and cycloalkanes (33.4%)^56^. However, diesel consists mostly n-alkanes (42.7%)^57^ and therefore it is expected a high abundance in alkane degradation enzymes in diesel contaminated soils. In fact, alkane 1-monooxygenase (EC:1.14.15.3), one of the most studied enzymes in hydrocarbon degrading bacteria, was detected in high abundance in these soils. Alkane monooxygenases are known key enzymes in aerobic degradation of alkanes by bacteria^58–60^. These enzymes hydroxylate alkanes to alcohols, which are further oxidized to fatty acids and catabolized via the bacterial β-oxidation pathway^61^.

In addition to alkane degrading enzymes, other enzymes in the fatty acid degradation pathway (ko00071) such as long-chain acyl-CoA dehydrogenase (EC:1.3.8.8) were also more abundant in diesel contaminated soils. Unlike diesel, which contains aromatic hydrocarbons, biodiesel consists of monoalkyl esters of long-chain fatty acids derived from renewable biolipids^62^. These fatty acid (m)ethyl esters are generally produced from natural oils or fats and it is expected a higher abundance of FAME degradation enzymes in biodiesel contaminated soils. This was true for rubredoxin-NAD + reductase (EC:1.18.1.1) and delta3-delta2-enoyl-CoA isomerase (EC:5.3.3.8). Rubredoxin-NAD + reductase is an important enzyme in the hydrocarbon hydroxylating system^63,64^ and several species of *Pseudomonas* such as *P. oleovorans*^65^, *P. oleovorans* and *P. putida*^66^ are known to produce this enzyme. Therefore, the dominance of *Pseudomonas* spp. in biodiesel profiles may be associated with a higher abundance of predicted Rubredoxin-NAD + reductase in these soils.

We also used PICRUSt2 to identify the taxa contribution of hydrocarbon degrading enzymes (Fig. 7B). Our analyses indicate a high contribution of members of the family Burkholderiaceae and the genus *Novosphingobium* in enzymes associated with benzoate degradation. Lyu et al.^67^ reported that *Novosphingobium pentaromativorans* US6-1 is able to degrade a large spectrum of aromatic hydrocarbons, ranging from monocyclic to polycyclic hydrocarbons. Most recently, Wang et al.^68^ conducted a genomic comparison analysis of 22 genomes of *Novosphingobium* strains and identified that they shared most degradative pathways including degradation of aromatic compounds and benzoate degradation. In our study, diesel contaminated soils had a higher abundance of *Novosphingobium* spp. (Figs. 6, S3), which suggest that aromatic hydrocarbons in diesel fuel are selecting for competent taxa do degrade these compounds. Moreover, most of predicted cyclohexane degradation (i.e., haloalkane dehalogenase EC:3.8.1.5) was attributed to the genera *Anaeromyxobacter* and *Rhodococcus*. As a facultative anaerobic myxobacterium, the presence of *Anaeromyxobacter* after a 1-year incubation suggests that natural attenuation has occurred under anoxic conditions. Our analysis revealed that sequences of *Rhodococcus* spp. not only contributed to predicted degradation of cyclohexenes but also in FAME degradation. For example, predicted alkane 1-monooxygenase (EC:1.14.15.3) was highly attributed to *Rhodococcus* spp., as multiple alkane hydroxylases have been identified as a common feature of this genus^39^. Although the presence of *Rhodococcus* spp. highly contributed to FAME degradation enzymes (i.e., EC:1.14.15.3 and EC:1.3.8.8), most of predicted contribution in this pathway was due to *Pseudomonas* spp. In biodiesel contaminated soils, we previously detected a higher abundance of *Pseudomonas* spp. (Fig. 6), which may suggest that the presence of long-chain fatty acid (m)ethyl esters in biodiesel fuel most likely selected for FAME degrading *Pseudomonas* spp. in these soils.

## Conclusions

This study assessed the impacts of diesel and biodiesel fuel on soil microbial activity within the first five weeks of contamination and shifts in microbial community structure after a 1-year incubation. We combined methods such as PLFA analysis to detect immediate changes in microbial community structure and high throughput 16S rRNA amplicon sequencing for a high-resolution taxonomic assessment. We found the highest microbial activity rates in biodiesel contaminated soils and shifts in microbial community structure. Long-term soil contamination led to an overall lower bacterial richness and diversity when compared to control samples while selecting for specific groups of microorganisms. A significant number of bacteria taxa in our dataset were unique to control soils, which supports the evidence of detrimental effects of hydrocarbon contamination to soil microbial diversity. Diesel contamination highly selected for *Anaeromyxobacter and Rhodococcus* spp., whereas a high abundance of *Pseudomonas* and *Bacillus* was found in biodiesel samples*.* Analysis of predicted hydrocarbon-degrading enzymes also revealed differences in functional profiles based on diesel and biodiesel chemical composition. Here, we identified potential key bacterial taxa in enhancing natural attenuation (i.e., Burkholderiaceae, *Novosphingobium Anaeromyxobacter*, *Pseudomonas* and *Rhodococcus*). Together, our analyses provide a detailed examination of soil microbial community activity and structure following exposure to anthropogenic recalcitrant hydrocarbons (e.g., diesel and biodiesel) thus confirming its potential adverse effects in soil health.

## Methods

### Soil collection

A Dark Brown Chernozem soil collected near Saskatoon, SK—Canada was used in the study. The upper and lower slopes included an Ardill Association (upper Apk) upper slope (Rego—low organic matter) and a low-slope (Eluviated—high organic matter) on a transect, respectively. Soils were air-dried, sieved to pass a 5 mm mesh and analyzed for nutrient contents including total nitrogen (TN), measured by dry combustion method using a LECO TruMac CNS Analyzer, total carbon (TC) and total organic carbon (TOC), measured according to Dhillon et al.^69^ using a LECO C-632 Carbon Analyzer. Soil organic Matter (OM) was analyzed using the dry-ash method^70^. Soil pH was measured in a 2:1 soil: water slurry. Soil available ammonium and nitrate were determined colorimetrically (660 and 520 nm, respectively) according to Laverty and Bollo-Kamara^71^. Available phosphorus and potassium were measured using a modified Kelowna extraction^72^ and available sulfate by a calcium chloride extraction^70^.

### Microcosm and microbial activity

Air dried soils (n = 2) were subjected to two treatments including (i) biodiesel and (ii) diesel (10 × 10^4^ L/ha), and (iii) untreated control, each replicated five times (total of 30). For the treatments amended with diesel or biodiesel, 100 g of soil were weighed and placed into a 200 cc plastic vial and 5.0 mL of each contaminant poured onto the soil. Deionized water was added to control and contaminated soils as required to ensure the moisture content (60% MHC) at field capacity. Treatments were incubated at room temperature in a 1.0 L Mason jars equipped with a septum for gas sampling and assessed weekly for five weeks using a modified CO_2_ evolution method by Anderson and Domsch^73^. After a 1-week incubation, a 20-cc headspace gas sample was withdrawn from the Mason jars using a 25-cc plastic syringe. Samples were analyzed on a Shimatzu GC-8A gas chromatograph equipped with a Porapak-Q column and thermal conductivity detector set at 45 and 60 °C, respectively^74^. After sampling, soils were also checked for moisture content deionized water was added if necessary and jars were left open for a few minutes to allow for re-oxygenation, sealed and re-incubated until the next sampling. The rate of CO_2_ evolution was expressed as µg of CO_2_·g of soil^–1^·day^–1^ calculated from the difference between each sampling week (1–5) and the initial week. After the microbial activity assessments, soils were incubated for 1-year at room temperature according to Ramirez et al.^75^ and Craine et al.^76^. Microbial community structure was determined after incubation by phospholipid fatty acid analysis (PLFA) and high-throughput 16S rRNA amplicon sequencing.

### PLFA analysis

PLFA analysis of soil samples was based on a modified protocol from Helgason et al.^77^. Soil samples were sieved, freeze-dried and ground with mortar and pestle to maximize lipid recovery. Fatty acids were extracted from 4.0 g of lyophilized, ground soil in a methanol/chloroform mixture and then dried down under constant N_2_ flow. Neutral-, glyco- and phospho-lipids were separated using solid phase extraction columns (0.50 g Si; Varian Inc. Mississauga, ON), sequentially eluted with chloroform (CHCl_3_), acetone ((CH_3_)_2_CO) and methanol (MeOH) respectively, and the phospholipid fraction dried under N_2_ flow. The phospholipid fraction was methylated using a solution of 1:1 methanol/toluene and methanolic potassium hydroxide (KOH) at 35 °C. After methylation, the resulting fatty acid methyl esters (FAMEs) were analyzed using a Hewlett Packard 5890 Series II gas chromatograph equipped with a 25 m Ultra 2 column (J&W Scientific). Peaks were identified using fatty acid standards and MIDI identification software (MIDI Inc., Newark, DE) and quantified based on the addition of a known concentration of the internal standard methyl nonadecanoate (19:0)^77,78^.

Microbial biomass was determined by biomarker abundance calculated based on the peak area detected for each fatty acid, relative to that of a known quantity of the internal standard. Biomarkers used to represent Gram-positive bacteria (G+) were i14:0, i15:0, a15:0, i16:0, i17:0, a17:0. For Gram-negative bacteria (G−), biomarkers used were 16:1 ω7t, 16:1ω9c, 16:1ω7c, 18:1ω7c, 18:1ω9c, cy17:0, and cy19:0^79^. Fungal biomass was evaluated using the PLFA biomarker 18ω2:6,9. Total biomass was calculated as the sum of all detected PLFAs^80^ (Total of 48), including 14 biomarkers and 34 general fatty acids (non-biomarkers). All biomass values were reported based on dry soil weight in units of nmol·g^−1^ soil derived from individual molecular weights of each fatty acid^77,81^.

### PLFA statistical analyses

Analysis of variance for PLFA biomarkers and total PLFAs was conducted using SAS v. 9.4 (https://www.sas.com). Non-metric multidimensional scaling (MDS) analysis of PLFA community composition (biomarkers and general fatty acids) was carried out using PCOrd v. 6.0^82^ (https://www.wildblueberrymedia.net/pcord). The PLFA data was transformed to log (mol% + 1) and Sørensen distance measure was selected using the autopilot slow and thorough analysis option^83,84^. A random starting point was used for initial analysis and then optimized in previous ordinations to achieve the lowest stress (expressed as Kruskal stress). The Monte Carlo test of significance and Multi-Response Permutation Procedure (MRPP) were subsequently used to test for differences between groups.

### DNA extraction

Total soil community DNA was extracted using the MoBio PowerSoil extraction kit (MoBio Laboratories Inc., Carlsbad, CA) following the manufacturer’s protocols. The DNA yield was quantified using Qubit Fluorometric Quantitation (Invitrogen) and in a SYBR Safe (Invitrogen) 1% agarose gel by comparison with a high DNA mass ladder (Invitrogen) using a Bio-Rad Gel Doc XR System (Bio-Rad Laboratories, Mississauga, ON).

### High-throughput 16S rRNA amplicon sequencing

To determine the diversity and bacterial community composition, DNA samples were submitted for high throughput sequencing at McGill University and Génome Québec Innovation Centre using Illumina technology. The primer set, and PCR protocol used are described in Edwards et al. (2007). Briefly, PCR amplifications were conducted using the 520F (5′-AGCAGCCGCGGTAAT-3′)/799R2 (5′-CAGGGTATCTAATCCTGTT-3′) primer set that amplifies the V4 region of the 16S rRNA gene. Amplicons with attached Illumina flow cell adapter sequences were added in Illumina MiSeq 2.0 platform in equimolar concentrations. Sample libraries were prepared according to the MiSeq reagent kit preparation guide (Illumina, San Diego, CA), and the sequencing protocol from Caporaso et al.^85^.

### 16S rRNA amplicon sequencing bioinformatics and statistical analysis

Sequence reads were analyzed using QIIME 2 v. 2019.1^86^ using QIIME 2 pipelines^87^ (https://qiime2.org). The raw forward and reverse sequences were quality-filtered using DADA2^88^. To remove noise from the data, the first 25 nucleotides were removed from the forward and reverse reads according to visual inspection of the quality of the reads. High-quality reads were down-sampled to the smallest sample size and classified (99% similarity) using the Silva 132 database. Alpha and beta diversity analyses were conducted using the QIIME 2 plug-in q2-diversity. Microbial community composition between groups were plotted in a principal coordinate analysis (PCoA) based on weighted and unweighted unifrac distances generated in QIIME 2. Statistical significance among groups (slope and treatment) was determined by the nonparametric statistical method ADONIS^89^ with 999 permutations. Heatmap and ternary plots were generated with R v. 3.6.0 (R Foundation for Statistical Computing; available at http://www.R-project.org) using the VEGAN package (v. 2.5-7)^90^ and ggtern (v. 3.1.0)^91^, respectively. Analysis of variance followed by Tukey post hoc test and Spearman's rank correlations were performed using SAS v. 9.4 (https://www.sas.com). PICRUSt2^92,93^ (https://github.com/picrust/picrust2) was used to generate functional predictions based on normalized 16S rRNA gene abundance levels. The functions of predicted metagenomes were categorized with the KEGG pathways database^94^ and statistical analysis were performed using STAMP v. 2.1.3^95^ (https://beikolab.cs.dal.ca/software/STAMP).

### Data deposition

Metagenomic datasets were deposited in the NCBI sequence read archive (SRA) under the submission ID SUB7149058. The metagenomic project can also be accessed in NCBI under Genome Project ID 393205 (accession PRJNA393205, http://www.ncbi.nlm.nih.gov/bioproject/393205).

## Supplementary Information

Supplementary Information.

## References

[1] Mnif I, Sahnoun R, Ellouz-Chaabouni S (2017). Application of bacterial biosurfactants for enhanced removal and biodegradation of diesel oil. Process Saf. Environ. Prot..

[2] Abioye OP, Pascucci S (2011). Biological remediation of hydrocarbon and heavy metals contaminated soil. Soil Contamination.

[3] Zarinkamar F, Reypour F, Soleimanpour S (2013). Effect of diesel fuel contaminated soil on the germination and the growth of *Festuca arundinacea*. Res. J. Chem. Environ. Sci..

[4] Ashnani MHM, Johari A, Hashim H, Hasani E (2014). A source of renewable energy in Malaysia, why biodiesel?. Renew. Sustain. Energy Rev..

[5] Bücker F (2011). Impact of biodiesel on biodeterioration of stored Brazilian diesel oil. Int. Biodeterior. Biodegrad..

[6] Hawrot-Paw M, Izwikow M (2015). Ecotoxicologial effects of biodiesel in the soil. J. Ecol. Eng..

[7] Restrepo-Flórez J-M, Bassi A, Rehmann L, Thompson MR (2013). Effect of biodiesel addition on microbial community structure in a simulated fuel storage system. Bioresour. Technol..

[8] Silva GS (2012). Biodegradability of soy biodiesel in microcosm experiments using soil from the Atlantic Rain Forest. Appl. Soil Ecol..

[9] Prosser JI (2015). Dispersing misconceptions and identifying opportunities for the use of ‘omics’ in soil microbial ecology. Nat. Rev. Microbiol..

[10] Hawrot-Paw M, Martynus M (2011). The influence of diesel fuel and biodiesel on soil microbial biomass. Pol. J. Environ. Stud..

[11] Lahel A (2016). Effect of process parameters on the bioremediation of diesel contaminated soil by mixed microbial consortia. Int. Biodeterior. Biodegrad..

[12] Nwankwegu AS, Orji MU, Onwosi CO (2016). Studies on organic and in-organic biostimulants in bioremediation of diesel-contaminated arable soil. Chemosphere.

[13] Woźniak-Karczewska M (2019). Effect of bioaugmentation on long-term biodegradation of diesel/biodiesel blends in soil microcosms. Sci. Total Environ..

[14] Caspi R (2018). The MetaCyc database of metabolic pathways and enzymes. Nucleic Acids Res..

[15] Lapinskiene A, Martinkus P, Rebzdaite V (2006). Eco-toxicological studies of diesel and biodiesel fuels in aerated soil. Environ. Pollut..

[16] Schiewer S, Horel A (2017). Biodiesel addition influences biodegradation rates of fresh and artificially weathered diesel fuel in Alaskan sand. J. Cold Reg. Eng..

[17] Schreier CG, Walker WJ, Burns J, Wilkenfeld R (1999). Total organic carbon as a screening method for petroleum hydrocarbons. Chemosphere.

[18] Nimmo M, Worsfold P, Alan Townshend CP (2005). Carbon. Encyclopedia of Analytical Science.

[19] Margesin R, Schinner F (1997). Bioremediation of diesel-oil-contaminated alpine soils at low temperatures. Appl. Microbiol. Biotechnol..

[20] Møller J, Winther P, Lund B, Kirkebjerg K, Westermann P (1996). Bioventing of diesel oil-contaminated soil: Comparison of degradation rates in soil based on actual oil concentration and on respirometric data. J. Ind. Microbiol..

[21] Nakatsu CH (2013). Microbial processes: Community analysis. Ref. Modul. Earth Syst. Environ. Sci..

[22] Margesin R, Hämmerle M, Tscherko D (2007). Microbial activity and community composition during bioremediation of diesel-oil-contaminated soil: Effects of hydrocarbon concentration, fertilizers, and incubation time. Microb. Ecol..

[23] Owsianiak M (2009). Biodegradation of diesel/biodiesel blends by a consortium of hydrocarbon degraders: Effect of the type of blend and the addition of biosurfactants. Bioresour. Technol..

[24] Quideau SA (2016). Extraction and analysis of microbial phospholipid fatty acids in soils. J. Vis. Exp..

[25] Frostegård Å, Tunlid A, Bååth E (2010). Use and misuse of PLFA measurements in soils. Soil Biol. Biochem..

[26] Ruess L, Chamberlain PM (2010). The fat that matters: Soil food web analysis using fatty acids and their carbon stable isotope signature. Soil Biol. Biochem..

[27] Davila S (2017). Actinobacteria: Current research and perspectives for bioremediation of pesticides and heavy metals. Chemosphere.

[28] Sutton NB (2013). Impact of long-term diesel contamination on soil microbial cummunity structure. Appl. Environ. Microbiol..

[29] Kersters K, Vos PDE, Gillis M, Swings J, Vandamme P, Dworkin M, Falkow S, Rosenberg E, Schleifer K-H (2006). Introduction to the *Proteobacteria*. The Prokaryotes: A Handbook on the Biology of Bacteria.

[30] Bell TH (2013). Predictable bacterial composition and hydrocarbon degradation in Arctic soils following diesel and nutrient disturbance. ISME J..

[31] Brzeszcz J, Kaszycki P (2018). Aerobic bacteria degrading both n-alkanes and aromatic hydrocarbons: An undervalued strategy for metabolic diversity and flexibility. Biodegradation.

[32] Elumalai P (2019). Role of thermophilic bacteria (*Bacillus* and, *Geobacillus*) on crude oil degradation and biocorrosion in oil reservoir environment. 3Biotech.

[33] Mitter EK, de Freitas JR, Germida JJ (2017). Bacterial root microbiome of plants growing in oil sands reclamation covers. Front. Microbiol..

[34] Bundy JG, Paton GI, Campbell CD (2002). Microbial communities in different soil types do not converge after diesel contamination. J. Appl. Microbiol..

[35] Korenblum E, Souza DB, Penna M, Seldin L (2012). Molecular analysis of the bacterial communities in crude oil Samples from two Brazilian offshore petroleum platforms. Int. J. Microbiol..

[36] Kim TJ, Lee EY, Kim YJ, Cho KS, Ryu HW (2003). Degradation of polyaromatic hydrocarbons by *Burkholderia cepacia* 2A–12. World J. Microbiol. Biotechnol..

[37] Revathy T, Jayasri MA, Suthindhiran K (2015). Biodegradation of PAHs by *Burkholderia* sp. VITRSB1 isolated from marine sediments. Scientifica (Cairo).

[38] Ramos DT, da Silva MLB, Nossa CW, Alvarez PJJ, Corseuil HX (2014). Assessment of microbial communities associated with fermentative-methanogenic biodegradation of aromatic hydrocarbons in groundwater contaminated with a biodiesel blend (B20). Biodegradation.

[39] Whyte LG (2002). Gene cloning and characterization of multiple alkane hydroxylase systems in *Rhodococcus* strains Q15 and NRRL B-16531. Appl. Environ. Microbiol..

[40] Lee M, Kim MK, Singleton I, Goodfellow M, Lee S-T (2006). Enhanced biodegradation of diesel oil by a newly identified *Rhodococcus baikonurensis* EN3 in the presence of mycolic acid. J. Appl. Microbiol..

[41] Bateman JN, Speer B, Feduik L, Hartline RA (1986). Naphthalene association and uptake in *Pseudomonas putida*. J. Bacteriol..

[42] Rentz JA, Alvarez PJJ, Schnoor JL (2004). Repression of *Pseudomonas putida* phenanthrene-degrading activity by plant root extracts and exudates. Environ. Microbiol..

[43] Shukor MY (2009). Isolation and characterization of *Pseudomonas* diesel-degrading strain from Antartica. J. Environ. Biol..

[44] Meyer DD (2014). Bioremediation strategies for diesel and biodiesel in oxisol from southern Brazil. Int. Biodeterior. Biodegrad..

[45] Taccari M, Milanovic V, Comitini F, Casucci C, Ciani M (2012). Effects of biostimulation and bioaugmentation on diesel removal and bacterial community. Int. Biodeterior. Biodegrad..

[46] Fosso-Kankeu E (2017). Adaptation behaviour of bacterial species and impact on the biodegradation of biodiesel-diesel. Braz. J. Chem. Eng..

[47] Lutz G, Chavarría M, Arias ML, Mata-Segreda JF (2006). Microbial degradation of palm (*Elaeis guineensis*) biodiesel. Rev. Biol. Trop..

[48] Holmes AJ (2000). Diverse, yet-to-be-cultured members of the *Rubrobacter* subdivision of the Actinobacteria are widespread in Australian arid soils. FEMS Microbiol. Ecol..

[49] Wollherr A (2011). Pyrosequencing-based assessment of bacterial community structure along different management types in German forest and grassland soils. PLoS ONE.

[50] Crampon M, Bodilis J, Portet-Koltalo F (2018). Linking initial soil bacterial diversity and polycyclic aromatic hydrocarbons (PAHs) degradation potential. J. Hazard. Mater..

[51] Wang L, Li F, Zhan Y, Zhu L (2016). Shifts in microbial community structure during in situ surfactant-enhanced bioremediation of polycyclic aromatic hydrocarbon-contaminated soil. Environ. Sci. Pollut. Res..

[52] van Beilen JB, Kingma J, Witholt B (1994). Substrate specificity of the alkane hydroxylase system of *Pseudomonas oleovorans* GPo1. Enzyme Microb. Technol..

[53] Mukherjee A (2017). Bioinformatic approaches including predictive metagenomic profiling reveal characteristics of bacterial response to petroleum hydrocarbon contamination in diverse environments. Sci. Rep..

[54] Ono K, Nozaki M, Hayaishi O (1970). Purification and some properties of protocatechuate 4,5-dioxygenase. Biochim. Biophys. Acta Enzymol..

[55] Fung HKH (2015). Biochemical and biophysical characterisation of haloalkane dehalogenases DmrA and DmrB in *Mycobacterium* strain JS60 and their role in growth on haloalkanes. Mol. Microbiol..

[56] Kang Y-S, Park W (2010). Protection against diesel oil toxicity by sodium chloride-induced exopolysaccharides in *Acinetobacter* sp. strain DR1. J. Biosci. Bioeng..

[57] Ramadass K, Megharaj M, Venkateswarlu K, Naidu R (2017). Ecotoxicity of measured concentrations of soil-applied diesel: Effects on earthworm survival, dehydrogenase, urease and nitrification activities. Appl. Soil Ecol..

[58] Moreno R, Rojo F, Rojo F (2017). Enzymes for aerobic degradation of alkanes in bacteria. Aerobic Utilization of Hydrocarbons, Oils and Lipids.

[59] Mitter EK, de Freitas JR, Germida JJ (2020). Hydrocarbon-degrading genes in root endophytic communities on oil sands reclamation covers. Int. J. Phytoremediat..

[60] Mitter EK, Kataoka R, de Freitas JR, Germida JJ (2019). Potential use of endophytic root bacteria and host plants to degrade hydrocarbons. Int. J. Phytoremediat..

[61] Rojo F (2009). Degradation of alkanes by bacteria: Minireview. Environ. Microbiol..

[62] Dincer K (2008). Lower emissions from biodiesel combustion. Energy Sources A Recov. Util. Environ. Eff..

[63] Miri M, Bambai B, Tabandeh F, Sadeghizadeh M, Kamali N (2010). Production of a recombinant alkane hydroxylase (*Alk*B2) from *Alcanivorax borkumensis*. Biotechnol. Lett..

[64] Schomburg D, Stephan D, Schomburg D, Stephan D (1994). Rubredoxin-NAD+ reductase. Enzyme Handbook.

[65] Eggink G, Engel H, Vriend G, Terpstra P, Witholt B (1990). Rubredoxin reductase of *Pseudomonas oleovorans*. J. Mol. Biol..

[66] Hagelueken G (2007). Crystal structure of the electron transfer complex rubredoxin rubredoxin reductase of *Pseudomonas aeruginosa*. Proc. Natl. Acad. Sci..

[67] Lyu Y, Zheng W, Zheng T, Tian Y (2014). Biodegradation of polycyclic aromatic hydrocarbons by *Novosphingobium pentaromativorans* US6-1. PLoS ONE.

[68] Wang J (2018). Comparative genomics of degradative *Novosphingobium* strains with special reference to microcystin-degrading *Novosphingobium* sp. THN1. Front. Microbiol..

[69] Dhillon GS, Amichev BY, de Freitas JR, van Rees K (2015). Accurate and precise measurement of organic carbon content in carbonate-rich soils. Commun. Soil Sci. Plant Anal..

[70] McKeague JA (1978). Manual on SOIL sampling and Methods of Analysis.

[71] Laverty DH, Bollo-Kamara A (1988). Recommended Methods of Soil Analysis for Canadian Prairie Agricultural Soils.

[72] Qian P, Schoenaru JJ, Karamanos RE (1994). Simultaneous extraction of available phosphorus and potassium with a new soil test: A modification of Kelowna extraction. Commun. Soil Sci. Plant Anal..

[73] Anderson JPE, Domsch KH (1978). A physiological method for the quantitative measurement of microbial biomass in soils. Soil Biol. Biochem..

[74] de Freitas JR, Schoenau JJ, Boyetchko SM, Cyrenne SA (2003). Soil microbial populations, community composition, and activity as affected by repeated applications of hog and cattle manure in eastern Saskatchewan. Can. J. Microbiol..

[75] Ramirez KS, Craine JM, Fierer N (2012). Consistent effects of nitrogen amendments on soil microbial communities and processes across biomes. Glob. Change Biol..

[76] Craine JM, Fierer N, McLauchlan KK (2010). Widespread coupling between the rate and temperature sensitivity of organic matter decay. Nat. Geosci..

[77] Helgason BL, Walley FL, Germida JJ (2010). Long-term no-till management affects microbial biomass but not community composition in Canadian prairie agroecosytems. Soil Biol. Biochem..

[78] Drenovsky RE, Elliott GN, Graham KJ, Scow KM (2004). Comparison of phospholipid fatty acid (PLFA) and total soil fatty acid methyl esters (TSFAME) for characterizing soil microbial communities. Soil Biol. Biochem..

[79] Macdonald LM, Paterson E, Dawson LA, McDonald AJS (2004). Short-term effects of defoliation on the soil microbial community associated with two contrasting *Lolium perenne* cultivars. Soil Biol. Biochem..

[80] Zelles L, Bai QY, Beck T, Beese F (1992). Signature fatty acids in phospholipids and lipopolysaccharides as indicators of microbial biomass and community structure in agricultural soils. Soil Biol. Biochem..

[81] Hynes HM, Germida JJ (2012). Relationship between ammonia oxidizing bacteria and bioavailable nitrogen in harvested forest soils of central Alberta. Soil Biol. Biochem..

[82] McCune, B. & Mefford, M. J. *Multivariate analysis of Ecological Data* (2011).

[83] Helgason BL, Walley FL, Germida JJ (2010). No-till soil management increases microbial biomass and alters community profiles in soil aggregates. Appl. Soil Ecol..

[84] McCune, B. & Grace, J. B. *Analysis of Ecological Communities* (2002).

[85] Caporaso JG (2010). QIIME allows analysis of high-throughput community sequencing data. Nat. Methods.

[86] Boylen E (2018). QIIME 2: Reproducible, interactive, scalable, and extensible microbiome data science. PeerJ Prepr..

[87] Caporaso JG (2011). Moving pictures of the human microbiome. Genome Biol..

[88] Callahan BJ (2016). DADA2: High-resolution sample inference from Illumina amplicon data. Nat. Methods.

[89] Anderson MJ (2001). A new method for non-parametric multivariate analysis of variance. Austral. Ecol..

[90] Oksanen, J. *et al.**Community Ecology Package ‘vegan’* (2020).

[91] Hamilton, N. *ggtern: An Extension to ‘ggplot2’, for the Creation of Ternary Diagrams* (2018).

[92] Douglas GM (2020). PICRUSt2 for prediction of metagenome functions. Nat. Biotechnol..

[93] Langille MGI (2013). Predictive functional profiling of microbial communities using 16S rRNA marker gene sequences. Nat. Biotechnol..

[94] Kanehisa M, Sato Y, Furumichi M, Morishima K, Tanabe M (2019). New approach for understanding genome variations in KEGG. Nucleic Acids Res..

[95] Parks DH, Beiko RG (2010). Identifying biologically relevant differences between metagenomic communities. Bioinformatics.

