# Supplementary material for: Impact of diesel and biodiesel contamination on soil microbial community activity and structure
Source: Sci Rep. 2021 May 25;11:10856. doi: 10.1038/s41598-021-89637-y (PMC8149423; doi:10.1038/s41598-021-89637-y)
Supplement: Supplementary file 1 — Supplementary Information. [file 41598_2021_89637_MOESM1_ESM.docx]

**Supplementary Information**

Impact of Diesel and Biodiesel Contamination on Soil Microbial Community Activity and Structure

Eduardo K. Mitter^1^; James J. Germida^2^; J. Renato de Freitas^2^

^1^Department of Food and Bioproduct Sciences,

^2^Department of Soil Science, University of Saskatchewan, 51 Campus Drive, Saskatoon, SK, Canada S7N 5A8.

Contributions: EKM conducted the experiments, analyzed the data and wrote the

manuscript. JRF carried out the microbial activity assays. EKM, JRF and JJG collaborated in the experimental design, provided critical feedback and helped shape the research, analysis and manuscript.

Competing financial interests: The authors declare no competing financial interests.

*Corresponding author: Dr. Eduardo K. Mitter, Department of Food and Bioproduct Sciences, University of Saskatchewan, Saskatoon, Canada.

e-mail: eduardo.mitter@usask.ca

Table S1: Soil Physical and Chemical proprieties of samples collected in an upper slope and lower slope positions.

| Soil | pH | Texture | OM | N | S | P | K |
| --- | --- | --- | --- | --- | --- | --- | --- |
|  |  |  | (%) | ------------------- (mg·Kg^-1^ of Soil) ----------------- | | | |
| Upper Slope | 7.33 | Sandy loam | <1.0 | 3.4 | 5.2 | 4.3 | 152 |
| Lower Slope | 6.99 | Loamy Sand | 1.3 | 4.8 | 6.4 | 6.2 | 237 |

**Table S2**: Analysis of variance of total organic carbon (TOC), inorganic carbon (IC), total carbon (TC) and total nitrogen (TN) by treatment and soil type. Different letters within columns indicate significant differences (Tukey HSD *p* < 0.05) (n=5) after one year of incubation.

| Soil | Treatment | TOC | IC | TC | TN |
| --- | --- | --- | --- | --- | --- |
|  |  | ----------------------------------------------- (%) ---------------------------------------- | | | |
| Upper Slope | Control | 0.8^e^ | 1.2^b^ | 2.0^d^ | 0.1^a^ |
|  | Diesel | 1.6^d^ | 1.8^a^ | 3.4^c^ | 0.1^a^ |
|  | Biodiesel | 5.3^b^ | 0.9^bc^ | 6.2^b^ | 0.1a |
| Lower Slope | Control | 1.8^d^ | 0.0^d^ | 1.8^d^ | 0.2^a^ |
|  | Diesel | 3.0^c^ | 0.7^c^ | 3.7^c^ | 0.2^a^ |
|  | Biodiesel | 6.9^a^ | 0.4^c^ | 7.4^a^ | 0.2^a^ |
|  | Slope (S) | <.001 | <.001 | 0.003 | <.001 |
| *p* value | Treatment (T) | <.001 | <.001 | <.001 | 0.266 |
|  | S* T | 0.063 | 0.004 | 0.001 | 0.886 |

**Table S3**: Analysis of variance for gram positive (G+), gram negative (G-) fungal biomarkers and total PLFAs by soil type and treatment (n=5) after one year of incubation. Different letters within columns indicate significant differences (Tukey HSD *p* < 0.05).

| Soil | Treatment | G+ | G- | Fungal | Total PLFAs | G+ | G- |
| --- | --- | --- | --- | --- | --- | --- | --- |
|  |  | ------------- (nmol·g^-1^ soil) ------------- | | |  | -------- (mol %) ------- | |
| Upper Slope | Control | 2.9^b^ | 2.0^b^ | 0.4^a^ | 10.0^d^ | 27.1^ab^ | 27.8^b^ |
|  | Diesel | 2.1^b^ | 3.4^b^ | 0.3^a^ | 11.7^d^ | 21.3^b^ | 35.6^b^ |
|  | Biodiesel | 0.8^b^ | 29.8^a^ | 0.5^a^ | 49.6^a^ | 1.7^c^ | 64.7^a^ |
| Lower Slope | Control | 3.5^b^ | 5.1^b^ | 0.5^a^ | 18.1^cd^ | 21.9^b^ | 32.5^b^ |
|  | Diesel | 7.1^a^ | 4.8^b^ | 0.6^a^ | 23.5^bcd^ | 34.9^a^ | 23.5^b^ |
|  | Biodiesel | 1.3^b^ | 26.4^a^ | 1.6^a^ | 44.2^a^ | 3.8^c^ | 69.6^a^ |
| *p* value | Slope (S) | <0.01 | 0.590 | 0.097 | 0.2736 | 0.149 | 0.776 |
|  | Treatment (T) | <0.01 | <.001 | 0.198 | <.001 | <.001 | <.001 |
|  | S* T | 0.019 | 0.681 | 0.318 | <.001 | 0.019 | 0.050 |

**Fig. S1**. Total PLFA extracted and from an upper and lower slope soil under three different treatments (control, diesel and biodiesel). Error bars represent standard deviations (n=5) after one year of incubation. Different letters indicate significant differences (Tukey HSD *p* < 0.05).

**
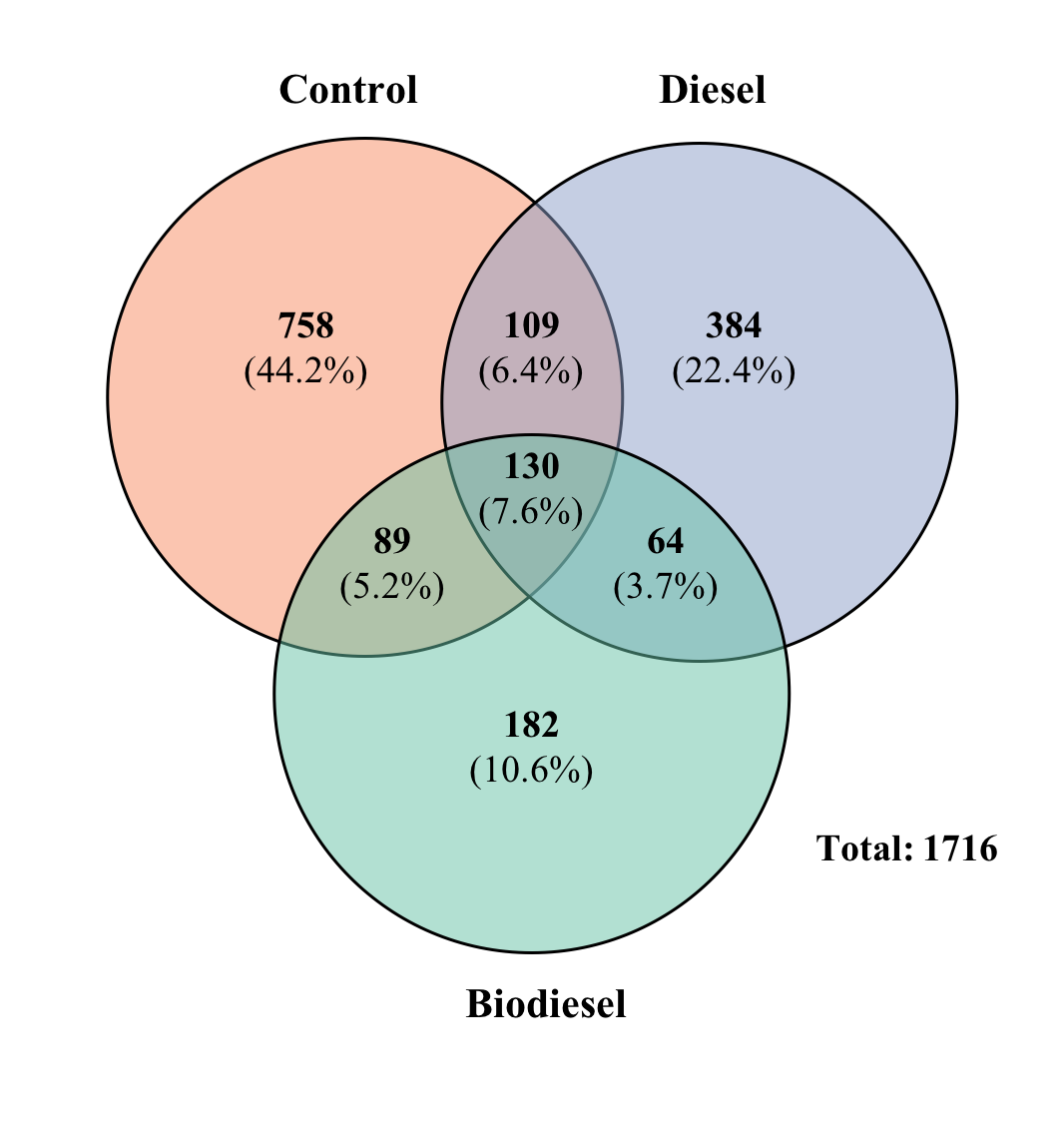
**

**Fig. S2.** Venn diagram of bacterial communities under three different treatments (control, diesel and biodiesel). Numbers indicated shared unique Amplicon sequence variants (ASVs) at 0.01 dissimilarity distances after removing singletons involved.

**Table S4.** Correlation indicating Spearman’s *r*_s_ values relating the most abundant bacterial phyla, class, order and soil parameters. NS = not significant and statistically significant correlations are indicated in bold type, * and ** denote *p* values < 0.05 and < 0.01, respectively. TOC = total organic carbon, IC= inorganic carbon, TC = total carbon, TN= total nitrogen.

|  | TOC | IC | TC | TN |
| --- | --- | --- | --- | --- |
| Top 5 phyla: |  |  |  |  |
| *Actinobacteria* | -0.58** | NS | -0.69** | NS |
| *Bacteroidetes* | -0.46* | NS | -0.59** | NS |
| *Firmicutes* | 0.48* | -0.55** | NS | 0.50* |
| *Gemmatimonadetes* | -0.74** | NS | -0.79** | NS |
| *Proteobacteria* | 0.50** | NS | 0.69** | NS |
| Top 10 classes: |  |  |  |  |
| *Actinobacteria* | -0.40* | NS | -0.41* | NS |
| *Alphaproteobacteria* | NS | NS | NS | NS |
| *Bacilli* | NS | NS | NS | NS |
| *Bacteroidia* | -0.77** | 0.46* | -0.58** | NS |
| *Clostridia* | 0.66** | -0.71** | NS | -0.53** |
| *Deltaproteobacteria* | NS | NS | NS | -0.56** |
| *Gammaproteobacteria* | NS | NS | NS | NS |
| *Gemmatimonadetes* | -0.47** | NS | -0.50* | NS |
| *Rubrobacteria* | NS | NS | NS | NS |
| *Thermoleophilia* | NS | NS | -0.52** | NS |
|  |  |  |  |  |
| Top 10 orders: |  |  |  |  |
| *Bacillales* | NS | NS | 0.46* | NS |
| *Betaproteobacteriales* | NS | NS | NS | NS |
| *Corynebacteriales* | NS | NS | NS | NS |
| *Enterobacteriales* | NS | NS | NS | NS |
| *Frankiales* | NS | NS | -0.41* | NS |
| *Myxococcales* | NS | NS | NS | NS |
| *Pseudomonadales* | NS | NS | 0.43* | NS |
| *Rubrobacterales* | NS | NS | NS | NS |
| *Solirubrobacterales* | NS | NS | NS | -0.43* |
| *Sphingomonadales* | NS | NS | 0.49* | NS |

**Table S5.** Correlation indicating Spearman’s *r*_s_ values relating diversity indexes and soil parameters. NS = not significant and statistically significant correlations are indicated in bold type, * and ** denote *p* values < 0.05 and < 0.01, respectively. TOC = total organic carbon, TC = total carbon, IC= inorganic carbon, TN= total nitrogen.

|  | TOC | TC | IC | TN |
| --- | --- | --- | --- | --- |
| Chao 1 | -0.56** | -0.66** | NS | NS |
| Shannon | -0.63** | -0.70** | NS | NS |
| Simpson | -0.63** | -0.69** | NS | NS |

**Fig. S3.** Extended bar plot representing statistically significant (Welch’s t-test, *p* < 0.05) bacterial genera abundance difference between soils amended with diesel and biodiesel. Genera shown represent at least 1% of the profile in one sample.

**Fig. S4.** PiCRUSt2 in silico functional analyses of contaminated (biodiesel and diesel) and control soils. Extended bar plots represent only statistically significant microbial pathways based on Welch’s t-test (*p* < 0.05) with the effect size greater than 10% and ratio greater than 2.0 between treatments.
